# Harnessing the Power of Metabolomics for Precision Oncology: Current Advances and Future Directions

**DOI:** 10.3390/cells14060402

**Published:** 2025-03-10

**Authors:** Manas Kohli, George Poulogiannis

**Affiliations:** Signalling and Cancer Metabolism Laboratory, Division of Cell and Molecular Biology, The Institute of Cancer Research, 237 Fulham Road, London SW3 6JB, UK; manas.kohli@icr.ac.uk

**Keywords:** metabolomics, cancer, precision medicine, therapy, metabolome

## Abstract

Metabolic reprogramming is a hallmark of cancer, with cancer cells acquiring many unique metabolic traits to support malignant growth, and extensive intra- and inter-tumour metabolic heterogeneity. Understanding these metabolic characteristics presents opportunities in precision medicine for both diagnosis and therapy. However, despite its potential, metabolic phenotyping has lagged behind genetic, transcriptomic, and immunohistochemical profiling in clinical applications. This is partly due to the lack of a single experimental technique capable of profiling the entire metabolome, necessitating the use of multiple technologies and approaches to capture the full range of cancer metabolic plasticity. This review examines the repertoire of tools available for profiling cancer metabolism, demonstrating their applications in preclinical and clinical settings. It also presents case studies illustrating how metabolomic profiling has been integrated with other omics technologies to gain insights into tumour biology and guide treatment strategies. This information aims to assist researchers in selecting the most effective tools for their studies and highlights the importance of combining different metabolic profiling techniques to comprehensively understand tumour metabolism.

## 1. Introduction

Metabolic reprogramming is undoubtedly one of the crucial hallmarks of cancer pathogenesis [1]. Tumour cells undergo several metabolic adaptations, often in a nutrient-deficient microenvironment, to sustain their increased rate of growth and proliferation [2]. Perhaps the most well-known example of cancer metabolic reprogramming is the ‘Warburg effect’, which refers to the tumour cells’ preference for using glycolysis even in the presence of oxygen (aerobic glycolysis) [3]. However, the switch from aerobic cellular respiration to aerobic glycolysis represents just the tip of the iceberg, and cancer cells acquire many other unique metabolic traits to support their malignant growth, including enhanced generation of antioxidant and detoxification capacity, upregulation of lipid metabolism or addiction to glutamine metabolism [4]. Tumours further display extensive intra and inter-tumour metabolic heterogeneity which is manifested in both spatial and temporal dimensions [5].

Understanding the metabolic characteristics and dependencies of cancer cells presents several opportunities in precision medicine from both a diagnostic and therapeutic perspective. Non-invasive metabolic profiling has been routinely applied to accurately detect and monitor tumour progression, whilst metabolic biomarkers show immense promise in identifying altered signalling pathways associated with specific cancer subtypes [6]. Despite promising applications, the use of metabolic phenotyping has lagged as a diagnostic and therapeutic tool compared to the more routinely used genetic or immunohistochemical profiling [7]. More studies are focused on profiling cancer genomes, transcriptomes, and proteomes, ignoring that the metabolome can often depict a more accurate and direct functional readout of the pathophysiological state of living systems [8].

This is also instigated by the fact that, unlike other omics profiling technologies, there is no singular experimental technique that enables the entire metabolome to be profiled at once [9]. As a consequence, it is rather challenging to capture the full range of metabolic plasticity cancer cells possess, limiting the identification and application of specific metabolic biomarkers. Given the vast chemical space, a cellular metabolome encompasses, metabolic profiling through multiple technologies and approaches is necessary to better understand and leverage tumour metabolic reprogramming. The purpose of this review is to examine the repertoire of tools available to researchers in tracking cancer metabolism to better aid them in decisions about which would be most effective for their studies. We also then proceed to demonstrate relevant examples of how these technologies have been applied both pre-clinically and in patient samples. Lastly, we demonstrate two case studies of how metabolomic profiling has been integrated with other omics technologies to gain better insights into tumour biology and guide treatment strategies in patients.

## 2. An Overview of Technologies and Approaches in Studying Tumour Metabolism

**Figure 1:**
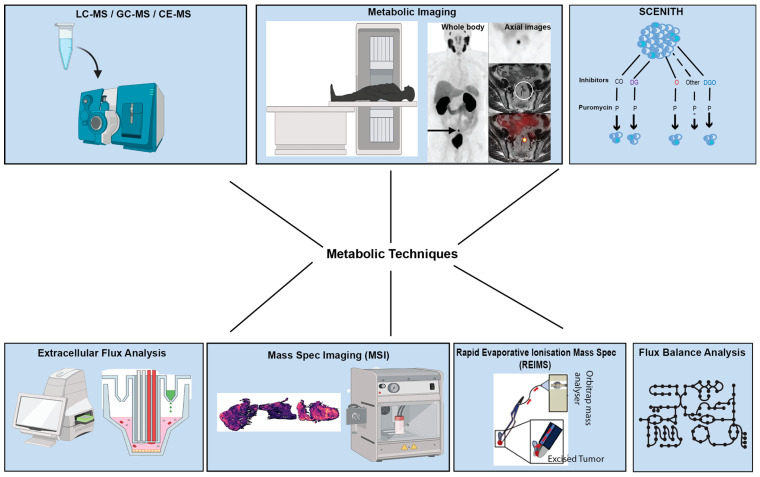
Overview of tools and technologies used to investigate metabolic phenotypes in cancer. Figure was created using images adapted from various publications [13,14,15] with BioRender.com.

A wide range of technologies and approaches are being utilised in studying tumour metabolism (Figure 1), starting from metabolomics, which refers to the set of analytical chemical techniques used to characterise the composition of biochemical mixtures [7]. Metabolomic techniques can be used to profile a wide range of metabolites from a variety of sources, including cells, tissues, and bodily fluids [10]. Unlike genomics, transcriptomics, and proteomics, metabolomics cannot be performed using a single instrument or technique because metabolites have a wide range of concentrations, sources, and chemical properties [9]. Another limitation of current metabolomic techniques is that it is often challenging to accurately capture the cell’s metabolic state by quenching all its metabolic pathways [11]. As a result, most metabolomic techniques focus on profiling and quantifying a smaller subset of pre-selected metabolites. Different metabolic profiling tools are often combined to take advantage of their strengths and compensate for their weaknesses [12]. This section will focus on these tools and how they are combined to more comprehensively profile tumour metabolism.

### 2.1. Nuclear Magnetic Resonance (NMR) Spectroscopy and Mass Spectrometric Methods Remain the Cornerstone of Metabolomic Profiling in Cancer

Over the past few decades, nuclear magnetic resonance (NMR) spectroscopy and separation techniques coupled with mass spectrometry have become standard practice in order to profile and quantify metabolites in biological samples. These methods have been extensively reviewed elsewhere [16,17]; hence, this section will only briefly address the workings of NMR, gas chromatography–mass spectrometry (GC-MS), liquid chromatography–mass spectrometry (LC-MS), and capillary electrophoresis mass spectrometry (CE-MS). NMR uses radiofrequency pulses and strong magnetic fields to analyse the environment of certain atomic nuclei, most commonly 1H and 13C [18]. In the strong magnetic field, these nuclei absorb and re-emit energy, producing characteristic signals that provide information about the chemical environment they are in. These spectra can then be used to estimate the relative concentration of specific metabolites or study the chemical environment of the analyte of interest. NMR can be applied in untargeted settings to quantify specific metabolic species, most commonly from 1H spectra [19]. However, these spectra can often be complex and difficult to analyse; computational techniques involving machine learning can help facilitate analyses and quantification of the metabolites [20]. More commonly, 13C NMR is used in targeted metabolomics settings for direct measurement of labelled carbons. Different tracers can be used to study specific metabolic pathways; for example, 13C1,2- glucose can be used to distinguish between oxidative and non-oxidative branches of the pentose phosphate pathway from differential labelling in downstream metabolites. 13C glutamine can be used to study glutaminolysis, while 13C-palmitic acid is often used to study fatty acid oxidation [17]. Metabolites can often be shuttled into many different pathways, but NMR allows the elucidation and quantification of metabolic flux into specific pathways due to the chemical environment of the labelled carbon, making it an attractive and powerful technique to use in metabolomics settings.

Despite its ease of use, NMR is limited in its ability to detect low-abundance metabolites and has lower sensitivity compared to mass-spectrometric techniques [21]. Mass spectrometry is most commonly coupled with three different separation techniques: gas chromatography, liquid chromatography, and capillary electrophoresis. The choice of separation technique often depends on the analyte and metabolic species to be determined [22]. GC-MS is best suited for detecting volatile compounds with low boiling points, such as free fatty acids [23]. GC-MS separation takes place inside capillary columns consisting of two phases—a stationary phase, coated on the inner surface of the column and a carrier gas with the vapourised metabolite sample, or the mobile phase. Each metabolite in the mobile phase interacts differently with the stationary phase of the column, depending on its chemical properties [24]. Metabolites are, therefore, separated based on their travel speed through the column. Gas chromatograms are often coupled to time-of-flight mass spectrometers (TOF-MS) that allow metabolite identification. Control of metabolite injection into the TOF analyser allows better detection of lower concentration metabolites and good resolution of the analytes in question [25]. GC-MS can detect certain volatile compounds with high precision, but it is limited to compounds that can easily be vaporised and are thermally liable. This represents a small subset of the cellular metabolome, limiting the applicability of GC-MS.

After the commercial introduction of atmospheric pressure ionisation sources, LC-MS gained more widespread use to profile metabolite mixtures [26]. Like GC-MS, LC-MS consists of a stationary solid phase and a mobile phase (which is a liquid in the case of LC-MS) inside a silica column. The mobile phase carries the metabolites to be analysed that interact and are eluted from the column depending on their chemical properties [26]. Advances in chromatographic separation techniques in LC-MS, such as hydrophilic interaction liquid mass chromatography (HILIC), have further led to better separation of compounds and resolution of peaks for LC-MS [27]. As a result, many complex metabolite mixtures can now be analysed using LC-MS with high accuracy.

CE-MS is a relatively new technique that is especially suited for the analysis of polar and charged metabolites in biological samples, such as nucleic acids, amino acids, peptides, and charged lipids [28]. Charged analytes migrate through a capillary that is placed in an electric field produced from electrodes. Analytes migrate according to their electrophoretic ability and are subsequently detected by a spectrometer. CE-MS has excellent separation for polar metabolites that may be structurally similar, but it possesses different mass:charge ratios and requires very little sample preparation. However, due to the relatively low use of CE-MS, challenges remain in its reproducibility and optimisation for specific metabolite classes [29].

Similarly to NMR, GC-, LC- and CE-MS can also be used in fluxomics applications to measure metabolic flux through specific reactions or pathways. This is achieved through radioisotope labelling of certain substrates, such as 13C-labelled glucose or 15N-labelled glutamine, and incorporation analysis of the labelled isotope into other compounds to track metabolic rewiring [30]. While mass spectrometry methods have been widely used, GC-, LC- and CE-MS are constrained in how many different species they can profile simultaneously and have difficulty detecting certain metabolites present at very low concentrations. Often, to profile many different metabolites, multiple different columns with different properties must be used, which can be quite expensive [10]. Furthermore, reproducibility issues in MS-based techniques often arise from variations in sample preparation, column performance, and instrument conditions, leading to inconsistent retention times and peak intensities. Additionally, differences in data processing methods, such as peak integration and normalisation techniques, can further compromise the comparability of results across experiments or laboratories. While NMR and mass-spectrometry methods may not provide a truly global metabolomic profile, they still nonetheless remain indispensable for detecting and quantifying metabolites. Many metabolomic strategies may use other techniques to profile cancer metabolism but still rely on the aforementioned technologies to validate findings.

### 2.2. Metabolic Imaging Enables the Examination of Metabolism In Vivo

As mentioned before, tumour metabolism can differ quite significantly from the metabolism of the surrounding normal tissue, given tumours’ high metabolic demands. This can be exploited through metabolic imaging that offers insights into altered biochemical processes characteristic of malignant growth [31]. While multiple imaging techniques exist, this section will address the two most widely used: positron emission tomography (PET) and magnetic resonance spectroscopy (MRS). PET provides detailed information about the metabolic activity of tissues in the body. It makes use of a radiotracer, most commonly 18F-fluorodeoxyglucose (FDG), that mimics glucose and is taken up in cells in a manner proportional to their metabolic activity [32]. Once 18F-FDG is taken up, it is phosphorylated by hexokinase to FDG-6 phosphate, which, unlike glucose, cannot be metabolised further, so it accumulates in tissues [33]. Once inside tissues, the tracer emits positrons, which, upon encountering electrons, emits photons that move in opposite directions. PET detectors that surround the body can detect these photons and use this information to infer the three-dimensional distribution of the tracer in the body [34]. This allows for a visualisation of metabolically active regions of the tissue, such as tumours, and can aid diagnosis and staging of tumours. PET is quite sensitive to changes in metabolism and can thus aid early cancer detection. It is also used to sensitively monitor the response of tumours to treatment, and because it can be used to scan the entire body, it can enable the detection and monitoring of metastases [35]. The biggest drawback of PET is its low spatial resolution for precisely locating tumours or fine details surrounding the tumour. PET is also limited in the types of species that can be used in radiotracing and the metabolites it can image in vivo [36]. Nonetheless, PET still has important applications in cancer diagnostics and detection with multiple Food and Drug Administration (FDA)-approved radiotracers used in the clinic.

MRS borrows from the workings of nuclear magnetic resonance to non-invasively profile tissues at the molecular level (see NMR section for further details). MRS is a versatile technique that provides quantitative data on metabolite concentrations in many different tissue types. However, it is limited by its spatial resolution and sensitivity as it requires metabolite concentrations to be on the order of millimolar (mM) [37]. MRS’s applications, however, have massively improved with the arrival of dissolution dynamic nuclear polarisation (DNP) that can enhance the sensitivity of 13C labelling by over 10,000 times. DNP hyperpolarises nuclear spins, which means that DNP 13C-labelled metabolites can be detected much more sensitively and at much lower concentrations than when using their non-hyperpolarised counterparts [38]. For the full details on the DNP MRS methodology, readers are referred to other relevant reviews [39].

While PET and MRS cannot be used to truly profile the metabolome of cancer, their importance is underscored by their application to profile metabolism in vivo, which can have important applications in precision medicine. These techniques are particularly useful when combined with other metabolomic methods, such as stable-isotope assisted LC-MS or MS imaging that can highlight important metabolites to examine in vivo.

### 2.3. Matrix-Assisted Laser Desorption/Ionisation (MALDI) and DESI Preserve Spatial Data During Metabolomic Profiling

While NMR, GC-MS, and LC-MS can provide quantitative measurements for a variety of metabolites, they utilise cell extracts from many cells and therefore, sacrifice spatial information for the corresponding metabolomics data. MALDI and DESI are both mass-spectrometry imaging (MSI) techniques that aim to profile many metabolites while allowing for spatial information preservation [40]. MALDI makes use of a matrix-like compound typically added onto a tissue segment or a biological extract [41]. The matrix is then irradiated with a laser, and it transfers the energy to the metabolites, leading to the desorption and ionisation of metabolites from the matrix. These ions are then accelerated into the mass analyser of a mass spectrometer for identification and relative quantification [42]. The laser desorption and ionisation process can be focused on a particular area of a slide, allowing for spatial information preservation [41]. MALDI is well suited for the analysis of small molecules, as well as those with low abundance. MALDI is also less harsh on the metabolite samples as they are not directly irradiated with the laser compared to some ionisation methods used in LC-MS [42]. Thus, MALDI is often adept in the analysis of labile compounds that can undergo fragmentation under harsher ionisation procedures.

DESI, on the other hand, can analyse the molecular composition of tissues directly without the need for external sample preparation. As it operates under atmospheric conditions, tissues in their native state can be analysed [43]. Instead of a matrix, DESI utilises a charged solvent, which contains ions used for desorption and ionisation, which are continuously sprayed onto the tissue surface. This spray forms small droplets upon hitting the sample surface, which interact with metabolites on the surface. This leads to a transfer of energy to the analytes on the surface, causing their desorption into the gas phase [44]. These desorbed molecules enter the gaseous phase and can then be ionised and analysed by a mass spectrometer. By moving the sample or DESI probe, spatial information can be generated for the tissue of interest [44]. DESI and MALDI excel at providing metabolomic profiling with spatial context, which is difficult to achieve with most other metabolomic techniques [45]. Advancements in technology have also allowed for their use with minimal sample preparation, increasing their applicability [46].

However, MALDI and DESI can only profile limited dynamic ranges of metabolites and struggle with the identification of isobaric compounds, limiting their more widespread use as metabolomic profiling technology [47]. LC-MS, on the other hand, makes use of chromatographic separation and so can handle broader ranges of metabolites [48]. Moreover, MALDI and DESI provide semi-quantitative metabolomics data, and given the lack of internal standards that are present in other mass-spectrometry-based techniques, they often face issues in reliably quantifying metabolites [49]. Technical issues with each technique, such as ion suppression effects (the presence of one analyte affects the ionisation of another) in MALDI, and polarity bias (where ionisation of certain compounds is favoured over others) in DESI, further limit the suitability of their use in metabolomic profiling [50]. Despite all these limitations, MALDI and DESI are incredibly powerful and versatile technologies as they provide spatially resolved metabolomics data for samples. With regard to precision medicine, this has important implications for studying the metabolic heterogeneity of individual tumour samples, the metabolic crosstalk between the tumour microenvironment and cancer cells, as well as the identification of drivers for metabolic phenotypes [47]. It is possible that MALDI and DESI could be used in clinical applications to identify metabolic phenotypes associated with drug response and identify tumour areas that may be more resistant to specific targeted therapies.

### 2.4. Extracellular Flux Analysis (EFA) Facilitates Investigation into Metabolic Phenotypes

Studying tumour metabolism involves examining not just metabolite levels and pathways but also the way cancer cells manage their energy sources via metabolic phenotypes. The Agilent Seahorse Extracellular Flux (XF) analyser is used to quantify “real-time” bioenergetic profiles of cells upon treatment with pharmacological agents [51]. It can simultaneously measure oxygen consumption rate (OCR) as a readout for mitochondrial oxidative phosphorylation (OXPHOS) activity and extracellular acidification rate (ECAR) that acts as an indicator of lactate secretion or glycolysis [52]. Importantly, the Seahorse instrument allows for the administration of up to four drugs, enabling the study of cellular metabolic adaptation under different conditions. An example application of EFA to fully characterise mitochondrial activity is through the common cell mito stress test, which starts out with a basal measurement of OCR for evaluating the basal respiration of cells [53]. The cells are then exposed to oligomycin, which greatly reduces mitochondrial respiration by inhibiting ATP synthase, causing a decrease in OCR. The second injection involves adding carbonyl cyanide 4-(trifluoromethoxy) phenylhydrazone (FCCP) to cells, which simulates maximal electron flow through the electron transport chain so that the respiratory chain operates at its maximum capacity. The difference between the maximal and the basal oxygen consumption is known as the spare respiratory capacity, which gives an indication of cells’ fitness and ability to respond to cellular stresses and different energetic demands. Lastly, rotenone and antimycin are injected into the media—these inhibit complex I and III, respectively, and shut down mitochondrial respiration, thereby allowing for the determination of non-mitochondrial respiration driven by processes outside the mitochondria. Different combinations of drugs can be used to test for various metabolic capacities and phenotypes of tumour cells in analogous ways [53].

EFA assays have enabled a more thorough investigation into the overall number of metabolic phenotypes. For example, when tumours exhibit gross phenotypic shifts due to, for example, the acquisition of specific driver mutations or the development of resistance to certain targeted therapies, they undergo major metabolic shifts, such as a change in a preferred carbon source or dependence on a particular nutrient. EFA assays are easy to perform and give a quantitative readout of various bioenergetic parameters; the ability to inject custom drugs also enables the testing of different metabolic vulnerabilities and dependencies [6]. However, care must be taken while performing these experiments and interpreting the results, as factors like media composition, cell density, and inhibitor concentration can all greatly affect the observed phenotypes. In addition, while OCR and ECAR give some measure of oxidative and glycolytic activity, many pathways contribute to these readings, and the contribution of secondary bioenergetic pathways cannot be ignored [54]. EFA assays offer a coarse interpretation of cellular metabolic shifts and are often used to accompany other signalling or metabolic findings but, nonetheless, represent useful accompaniments when exploring metabolic shifts and novel vulnerabilities [55].

### 2.5. SCENITH Can Be Used for Immuno-Metabolic Profiling Applications

In vitro EFA assays typically use many cells in bulk and specific growth media to perform the assays. They are unable to profile heterogeneous populations of cells and classify rare sub-populations of cells, and the change in media can lead to artefacts. Single-cell energetic metabolism by profiling translation inhibition (SCENITH), is a technique that allows single-cell energetic profiling of multiple cell types ex vivo and allows shifts in metabolism in certain sub-populations of cells to be detected upon certain events [56]. SCENITH allows the study of metabolic responses by measuring protein synthesis rates as a proxy for cellular metabolism. Protein synthesis rates can be reliably measured using the incorporation of puromycin and show a strong correlation with metabolic rates. Samples will typically be incubated with puromycin for a short time to allow labelling. Using a novel anti-puromycin monoclonal antibody, SCENITH then leverages cell sorting technologies such as fluorescence-activated cell sorting (FACS) to measure the incorporation of puromycin at a single-cell resolution. Similarly to EFA assays, SCENITH uses a combination of different metabolic inhibitors along with puromycin to study the contribution of different energetic pathways for specific cell types. An example of this may be incubating the cells with puromycin and 2-deoxy-d-glucose to study the energetic contribution of glycolysis [56].

SCENITH’s primary use has been for immuno-metabolic profiling as it allows for metabolic profiling of different cell types in the tumour microenvironment at single-cell resolution [56]. The identification of immune cells has been heavily optimised in FACS experiments, so SCENITH complements existing technologies to better understand the tumour microenvironment. This has especially important applications in improving the efficacy of immunotherapy. It cannot only help in the understanding of why certain patients respond, but also in finding potential ways to reprogram the metabolism of the microenvironment to improve response to immunotherapies [6]. SCENITH can also be performed ex vivo and so can be directly applied to patient serum samples, bypassing artefacts induced by culturing in non-physiological media that are commonly used in vitro. Despite this, SCENITH has some drawbacks in energetically profiling cells. SCENITH relies on measuring protein synthesis, which is an indirect measurement of cellular metabolism as opposed to the more direct measurements of OCR and ECAR used in EFA assays. SCENITH also relies on cellular fixation and cannot analyse energetic profiles in real-time in response to certain inhibitors like EFA assays [56]. Nonetheless, the primary draw of SCENITH that most other technologies fail to provide is single-cell energetic profiling. Given how heterogeneous the immune cell population is in the tumour microenvironment, SCENITH is emerging as a valuable tool to understand the numerous interactions tumour cells have with immune cells and how energetic profiles of immune cells govern their phenotypic behaviour. When combined with single-cell RNA-seq (scRNA-seq), SCENITH holds immense promise for improving the efficacy of immunotherapies [55].

### 2.6. Rapid Evaporative Ionisation Mass Spectrometry (REIMS) Permits Real-Time Analysis of Surgical Samples

A key feature that distinguishes metabolism from other cellular processes is its ability to be analysed immediately. Once a sample is ready to be analysed by a mass spectrometer, the instrument can generate a spectrum and identify certain metabolites within a short duration. This is in direct contrast, for instance, to profiling tumour genomics, as even after a tedious process of DNA extraction, it takes significantly longer to sequence and identify mutations. The entire process from sampling to mass detection is shortened by recent technological advancements coupling surgical techniques to metabolomic profiling through rapid evaporative ionisation mass spectrometry (REIMS) [57]. REIMS allows near-instantaneous metabolic profiling of tissues, enabling surgeons to identify boundaries between tumours and surrounding non-cancerous tissue. REIMS is typically used alongside an electrosurgical knife that cauterises tissue during surgery. As the knife cauterises the tissue, it generates an aerosol known as the electrosurgical plume, which contains small particles of the tissue. The electrosurgical plume is directed into the REIMS ionisation source where the tissue particles are subjected to an electric field and heated vaporisation chamber. The high temperature in the chamber causes the particles to rapidly evaporate, leading to the lipids in the tissue being ionised, allowing for them to be analysed by mass spectrometry [58]. Given that cancerous and non-cancerous tissues have distinct metabolic profiles, REIMS allows for differentiation between the two. Importantly, it has also been shown that distinct metabolic signatures can be linked to specific oncogenic drivers; for example, *PIK3CA* mutant tumours display unique metabolic profiles characterised by enhanced arachidonic acid levels [59]. In addition to identifying tumour boundaries, this allows for REIMS to provide a near real-time diagnosis of tumours driven by specific oncogenic drivers [59], opening therapeutic opportunities during surgery even for regions of the tumour that cannot be fully surgically excised.

### 2.7. Flux Balance Analysis (FBA) Allows a near Genome-Wide View of Cancer Metabolism

While it is difficult to globally profile the metabolome with current experimental methods, computational methods can aid in predicting cellular metabolomes. One such method is flux balance analysis (FBA), which analyses the flow of metabolites through metabolic networks [60]. FBA calculates metabolite flow using genome-scale metabolic network reconstructions curated over the past few decades, including Recon1/2/3D [61], HMR1/2 [62] and the most recent Human1 [63]. It operates under the assumption that cellular metabolic processes seek a steady state with fluxes through metabolic reactions optimised to reach certain cellular objectives such as maximisation of growth, maintenance of energy levels represented through ATP:ADP ratios or maximising a cell’s reductive potential to protect against harmful reactive oxygen species. FBA utilises linear programming to predict optimal flux distributions through minimising or maximising objective functions subject to constraints of a predefined stoichiometric model of the metabolic network. These flux distributions can then be interpreted as a surrogate for metabolic activity through a particular reaction or pathway.

FBA’s real value lies in being able to set appropriate constraints on the metabolic network from available transcriptomics or proteomics data—this allows unique solutions for flux vectors to be generated depending on the sample of interest. In this way, FBA can model, for example, different cancer cell lines or patients and identify key metabolic pathways influencing metabolic phenotypes. In addition, FBA can also be used to predict gene essentiality through in silico knockout of genes and metabolic pathways, though it must be noted that these are limited to those present in the model. Those that lead to a decrease in the value of the objective function serve as a readout for a loss in viability, potentially unveiling actionable targets [63]. FBA has been used routinely in microbial settings, particularly in industrial applications involving metabolic engineering [64]. While FBA is not commonplace in studying human metabolism, advances in model simulation methods and curation are improving the accuracy of its use in studying cancer [63]. FBA holds great promise as a companion technique in metabolomics offering a quantitative framework to reduce the metabolic space needed for study. This can allow focus on a few select reactions or pathways through experimental methods such as LC-MS-based metabolomics.

### 2.8. Comparison of Different Metabolomics Techniques

The advantages and disadvantages of the aforementioned technologies used to investigate cancer metabolism are summarised in Table 1.

## 3. The Application of Metabolomics in the Diagnosis and Treatment of Cancer

**Figure 2:**
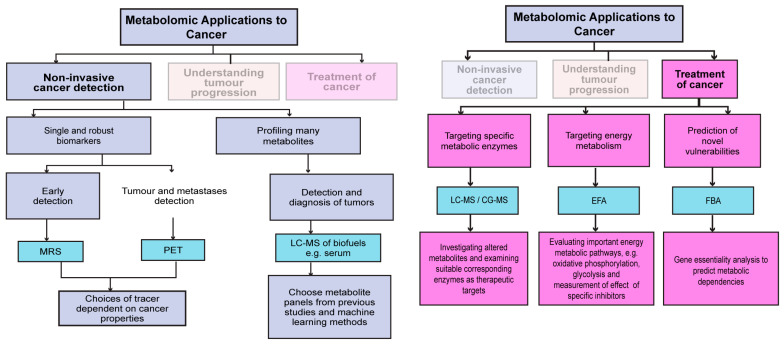

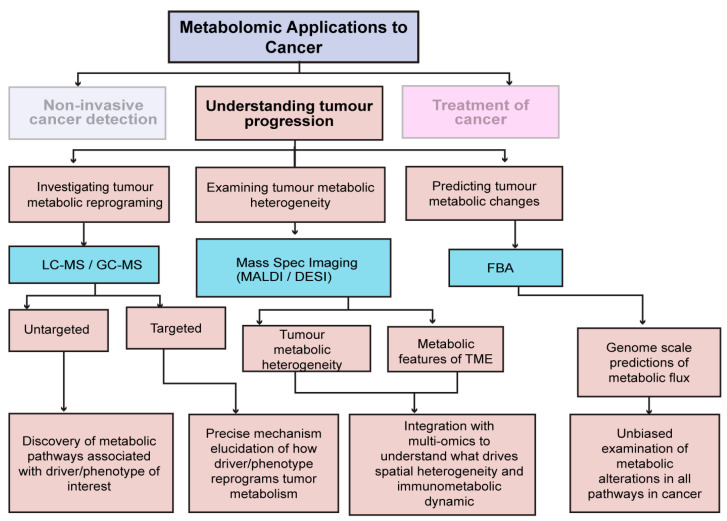
Describing how different metabolic tracking tools and methodologies fit in with different applications for detecting, understanding and treating cancer.

This section covers some key applications of the aforementioned metabolomics techniques in cancer research, in particular, how metabolomics can aid in tumour detection, understanding tumourigenesis, monitoring response to therapies, identifying novel metabolic vulnerabilities, and improving the outcome of existing therapies (Figure 2).

### 3.1. Non-Invasive Metabolic Tracking for Tumour Detection and Response to Therapy

Tumour metabolic reprogramming often occurs downstream of signalling pathways, perturbations, or other important regulators. Metabolomics can detect specific metabolites whose levels are altered throughout neoplastic progression, providing effective biomarkers for cancer diagnosis and disease monitoring [6]. In particular, non-invasive metabolic tracking has attracted significant attention due to its simplicity and increased patient compliance compared to obtaining biopsies to profile cancers. Non-invasive metabolic tracking tools in clinical settings usually comprise metabolic imaging of selective biomarkers or direct metabolomics analysis of biofluids [65]. While many different types of samples can be used for the latter analysis, such as urine, saliva, faecal matter, and breath, this review will examine a few brief examples using serum and plasma, and the reader is referred to other reviews for more comprehensive coverage of this topic in different cancer subtypes [66,67,68,69,70,71].

Imaging techniques, including magnetic resonance imaging and PET, are widely used in the clinic for the detection and diagnosis of cancer. As mentioned in the previous section, one of the most common and first tracers used for PET imaging was 18F-FDG-PET, which takes advantage of a tumour’s enhanced metabolic activity and glucose uptake. Its application extends to initial diagnoses, including the estimation of staging, lymph node involvement, and the presence of metastases. It is also used after treatment to assess responses to therapy and follow-up recurrent cases [72]. Due to its high retention within cells and high rate of clearance from the body, 18F-FDG-PET is used to diagnose many different types of cancers, including lung, head and neck, ovarian, and breast cancers, among others [73]. The 18F-FDG-PET is used in guiding treatment strategies for pancreatic cancer where it has been applied to aid in choosing between curative and palliative approaches to treatment [74]. However, 18F-FDG may not always be the most appropriate tracer for PET imaging. Some tumours, such as prostate cancers, have low glucose avidity, and instead rely more on fatty acid oxidation [75], while in other tumours, such as gliomas, the high background of the surrounding neural tissue can interfere with detection [76]. Other tracers that examine different metabolic pathways or substrates can be used instead. For example, L-[5-11C]-glutamine [77], [18F]-(2S,4R)-4-fluoroglutamine, and [18F]-(2S,4S)-4-(3-fluoropropyl)glutamine [78] have been used to study glutaminolysis, which has been shown to be significantly altered in some cancers. [18F]-(2S,4R)-4-fluoroglutamine has been applied to a recent glioma clinical trial, as gliomas show higher rates of glutaminolysis [79]. Other tumours rely more on lipid metabolism and show increased lipid species synthesis; this idea has been exploited to develop [18F]-fluoromethylcholine and [18F]-fluoroethylcholine, which label the phospholipid head group choline and can be used as a proxy for membrane synthesis [80,81]. These tracers are currently used in the clinic to image prostate cancer. The use of different metabolic species makes PET a very versatile and useful clinical tool for cancer diagnosis.

Magnetic resonance spectroscopy imaging (MRSI) is another invaluable imaging modality that allows for the detection of metabolites spatially resolved in vivo, and unlike PET, it does not involve exposure to ionising radiation. Early studies using 1H and 13C MRSI had some limitations in their applications due to low spatial resolution, but rapid advances have been made with the advent of DNP [82]. One of the first and most widely used molecules to be hyperpolarised for MRSI is [1-13C] pyruvate [83]. The hyperpolarised 13C from [1-13C] pyruvate can shift its environment if it is converted to lactate, catalysed by lactate dehydrogenase, and serves as a proxy for measuring pyruvate’s entry into the mitochondria and the TCA cycle. Due to its sensitive detection, hyperpolarised [1-13C] pyruvate has been used in preclinical studies to show that lactate labelling can be used to assess tumour grade and early treatment response [84,85]. It has been applied to clinical studies of breast, prostate, brain, renal, and pancreatic cancers, and has been used to assess response to immune checkpoint inhibitors in a prostate cancer patient [86]. MRSI of lactate labelling from hyperpolarised [1-13C] pyruvate can also identify tumour metabolic subtypes and gain insights into likely responses to therapy. For example, in a study conducted on orthotopically implanted patient-derived glioblastoma xenografts, the levels of lactate labelling corresponded to transcriptomic-derived subtypes [87]. High levels of lactate labelling resembled the mesenchymal subtype that was more radioresistant [88] compared to tumours that showed lower levels of lactate labelling and resembled more oxidative neural progenitor like glioblastomas [87]. While hyperpolarised [1-13C] pyruvate has been more widely used clinically, other emerging hyperpolarised metabolites, such as [1,4-13C2] fumarate for imaging necrotic cell death [89], are also increasingly being used in various applications.

While PET and MRSI have been applied quite extensively in clinical contexts, they are not without drawbacks. FDG-PET, for example, is often useful for diagnosing later, but not early-stage cancers. This is because 18F-FDG is a substrate for facilitated glucose transporters (GLUTs) but not sodium-dependent glucose transporters (SGLTs), the former of which are often more highly expressed in later-stage tumours [90]. Moreover, whilst FDG-PET has been widely applied to monitor cancer therapy response, it has shown low accuracy in the context of some targeted therapies. Inflammatory phenotypes, in particular, have been well documented to compromise early assessment of response. An example that exemplifies this are melanomas, which when treated with anti-CTLA4 or anti-PDL1 immunotherapies, can often show heightened immune responses that would be detected on a PET scan as high FDG uptake. This suggests it would be harder to differentiate tumour relapse from response to immunotherapy [91]. FDG-PET also has limited applicability in studying certain tumours and metastases that are surrounded by tissue with high glucose uptake. Continuing on from the melanoma example, a common site of metastases is to the brain that shows the highest uptake of glucose in the body, making it difficult for detection using FDG-PET. MRSI has the potential to improve detection and evaluate treatment response in brain metastases through evaluation of metabolite concentrations such as choline, creatine, and N-acetylaspartate that change in tumours due to treatment [92]. While MRSI can aid in overcoming some of PET’s limitations, the lack of standard procedures and difficulty in the analysis of spectra, as well as the fact that hyperpolarised metabolites can only retain their polarised state for 20–30 s, limiting its applicability in the clinic [92]. Both PET and MRSI are also limited in the species they can measure and fail to capture the whole tumour metabolome.

Compared to imaging techniques, the analysis of biofluids allows for the convenient and sensitive profiling of a greater diversity of metabolic species. As a result, there have been efforts to use serum and plasma-derived metabolic biomarkers to detect cancer and monitor response. For specific tissue types of cancer, specific serum markers have been associated with tumour detection. Examples include L-octanoylcarnitine, 5-oxoproline, hypoxanthine, and docosahexaenoic acid for breast cancer [93] and glutamate, choline, 1,5-anhydro-D-glucitol, betaine, and methylguanidine, among others. Another study focused on the presence of free amino acids in plasma and highlighted consistent biomarkers among the many different types of cancer, including lung, gastric, breast, colorectal, and prostate cancers, compared to the matched, healthy control specimens [94].

Although these studies have revealed potential biomarkers for diagnosis, they are quite limited in their cohort size; they lack independent cohorts for validation and offer limited reproducibility due to the differences in how metabolites are extracted and profiled between studies. For non-invasive biofluid metabolomics studies, variability in sample collection and analysis has partly been addressed by standardising pre-analytical factors, such as patient fasting status, collection time, and storage conditions. Additionally, statistical normalisation and quality control measures have been applied to enhance reproducibility and ensure accurate biomarker identification [6]. In addition, recent studies have utilised metabolomics, which measure the levels of many metabolites coupled with machine learning methods to identify subsets of metabolites whose levels can have diagnostic value. One recent example comes from Chen et al., who analysed plasma metabolite levels of 147 metabolites from 702 patients comprising three distinct cohorts from a multi-centre study [95]. Using least absolute shrinkage and selection operator (LASSO) regression to select the most relevant metabolites coupled with random forest classifiers, the authors were able to develop a diagnostic model based on 10 metabolites that accurately differentiated gastric cancer samples from healthy patients for all stages, including stage 1A. Using a similar method, they also developed a prognostic model based on 28 metabolites that could predict prognosis and survival at different stages. Importantly, these models were able to outperform models that use existing clinical characteristics, highlighting the usefulness of plasma metabolomics as a diagnostic and prognostic tool.

### 3.2. Metabolomic Profiling of Clinical Specimens to Understand Tumour Progression

Non-invasive profiling of tumours has great diagnostic and prognostic value, but it has limited value in being able to understand tumour metabolic heterogeneity, and the relationship between the tumour’s genome, transcriptome, and metabolome. It also has limited value for discovering metabolic vulnerabilities that can aid therapeutic targeting. When surgical specimens become available, the metabolomic analysis of tumours and adjacent normal tissues allows for a more thorough understanding of metabolic reprogramming and metabolic interactions that occur between tumours and their microenvironment. Mass spectrometry-based metabolomics have been applied widely for this purpose, and relevant examples are reviewed in Section 4; this section will briefly examine applications of MSI.

MSI’s major power comes from preserving spatial metabolomic information and the ability to integrate other spatial multi-omic data, such as spatial genomics and transcriptomics. The latter has been exemplified by studies that investigated Myc metabolic reprogramming in breast cancer. Using DESI MSI analysis of tumours derived from inducible models of Myc, Kreuzaler et al. were able to demonstrate that the upregulation of Myc led to higher levels of pantothenic acid, a precursor to coenzyme A, which is involved in a myriad of metabolic processes [15]. Importantly, they were able to demonstrate in tumours with heterogeneous Myc expression that pantothenic acid levels followed a distribution similar to the expression patterns of Myc. Importantly, this Myc-associated metabolic biomarker was missed when looking at the bulk metabolic analysis of Myc heterogeneous tumours, highlighting the spatial information preserved by DESI. In a separate study [91], DESI was also able to show spatiotemporal changes in metabolism that were associated with Myc amplification, which led to lipogenesis induction through SREBP cooperation. This led to increases in phosphatidylglycerol (PG) species, altering membrane and mitochondrial functions.

Perhaps the most relevant application of DESI in recent times is its ability to study spatial metabolic heterogeneity and the interaction of tumour cells with the TME, as shown in a recent study on gastric cancer. Sun et al. revealed significant intratumor heterogeneity, identifying distinct tissue regions with varied metabolite, lipid, and gene expression signatures [96]. Notably, they observed stepwise metabolic alterations along the progression from normal epithelium to a serrated lesion to a tumour, shedding light on the underlying metabolic shifts driving gastric cancer development. Furthermore, the study highlighted the immunometabolic dynamics at the tumour interface region, where immune and inflammation-related signatures are prominent. By mapping the spatial transcriptome, the researchers identified a distinct “interface” cluster rich in immune cell populations, particularly plasma B cells and follicular B cells. Tumour cells at this interface were characterised by alterations in glutamine metabolism and increased levels of long-chain unsaturated fatty acids. The upregulation of genes involved in FA synthesis and arachidonic acid metabolism in PLT indicated an enhanced inflammatory response, potentially influencing immune cell functions and tumour cell proliferation. Overall, this study shows how MSI can provide comprehensive insights into the spatial heterogeneity and immunometabolic dynamics of cancer, unveiling novel molecular signatures and potential therapeutic targets.

### 3.3. Metabolomics Applied to Therapeutic Settings

Metabolic rewiring by cancer cells has been extensively studied and exploited to develop therapeutics to better treat tumours. This section briefly focuses on applications of metabolomics techniques to discover metabolic vulnerabilities or targets that can be used to improve existing therapies. For a more thorough review of metabolic therapies in cancer, readers are referred to the following excellent reviews [65,97,98].

LC-MS- and GC-MS-based methods have predominantly been used to identify potential metabolic targets for therapy and have been successfully applied to the clinic. Perhaps the most classic example is the development of IDH-1/2 mutant inhibitors used to treat acute myeloid leukaemia (AML) and gliomas. In IDH-1/2 mutant AML tumours, LC-MS revealed that there was the accumulation of the D-2HG oncometabolite, supporting malignant transformation [99]. Targeting the mutant IDH-1/2 enzyme through Ivosidenib and Enasidenib greatly limited proliferation in these tumours, and it was subsequently approved by the FDA [100]. Since then, LC-MS has been used to discover various metabolic targets for therapy, including enzymes involved in nucleotide synthesis, redox metabolism, and amino acid metabolism. Further examples are examined in Section 4.

EFA methods can also be used to uncover relevant metabolic therapies pertaining to energy metabolism. Metabolic pathways related to the generation of ATP have been shown to be important not just for cancer cell’s energetic needs, but also for their signalling, differentiation, invasion and ability to metastasise. Accordingly, inhibitors of glycolysis [101], fatty acid metabolism, glutaminolysis, the TCA cycle and oxidative phosphorylation (OXPHOS) have been developed [102], which can be used to directly target tumours or sensitise them to existing therapies as part of combination therapy strategies [103]. EFA methods, such as the Seahorse assay, can unveil the metabolic pathways tumours primarily rely on and expose relevant metabolic vulnerabilities. An example of this was applied in studying NRAS-mutated melanoma, which lacks any clinically approved targeted therapy [104]. The authors were able to use the Seahorse assay to show that NRAS-mutated melanoma cells were dependent on glucose and showed low fuel flexibility, even under glucose deprivation. This was followed up by showing that NRAS mutant tumours activated BRAF signalling that led to the phosphorylation of 6-phosphofructo-2-kinase/fructose-2,6-bisphosphatase-2/3 (PFKFB2/PFKFB3) and allosteric activation of PFKM1. This led to a positive feedback cycle coupling glycolytic flux to RAS signalling. Subsequently, inhibition of glucose metabolism through a combination of 2-deoxy-D-glucose (2-DG) and sorafenib disrupted this cycle and inhibited tumour growth in patient-derived xenografts. These studies illustrate how energy metabolism through EFA can be used to inform on appropriate metabolic therapies to treat certain tumour subsets.

## 4. Metabolomics in Multimodal Profiling of Clinical Cohorts

Advancements in metabolomics technologies have now allowed for better profiling of patients’ metabolomes. However, many clinical trials still omit profiling cancer metabolomes, often choosing to focus on other cellular profiles, such as the genome, transcriptome, or proteome [8]. Nevertheless, recent studies have shown that multimodal approaches that utilise metabolomics carry great diagnostic and prognostic potential in understanding tumour progression and identifying key metabolic vulnerabilities in patients. While it is not possible to review all these studies, this review will focus on two specific case studies and how metabolomics have been applied to patient cohorts to better understand the underlying cancer biology. The first concerns triple-negative breast cancer in the Fudan cohort, and the second studies a glioblastoma (GBM) cohort.

### 4.1. Fudan Cohort

Breast cancers are histologically characterised by the expression of receptor subtypes. Tumours not expressing the oestrogen receptor (ER) or progesterone receptor (PR) and have no human epidermal growth factor receptor 2 (HER2; also referred to by its gene name ERBB2) amplification are classified as triple-negative breast cancer (TNBC) [105]. TNBCs typically make up 10–20% of most breast cancer cases and are biologically more aggressive, often being associated with the worst five-year prognosis, with higher rates of early distant recurrence [106]. They are among the most heterogeneous subtypes of cancers, with different cases presenting various drivers, and they lack more obvious targets, such as the ER or HER2 receptors [107]. Because of the challenges in targeting TNBCs, there are limited treatment options. While there are some exceptions, such as the use of Olaparib [105] and an immune checkpoint inhibitor, Atezolizumab [108], for patients with germline mutations in BRCA genes and PD-L1+ tumours, respectively, there are relatively few approved targeted therapeutics for the treatment of TNBCs, with chemotherapy still remaining as the standard of care. Therefore, there is an urgent need to identify target pathways for TNBCs.

To better understand the heterogeneity of the TNBC landscape and to find actionable targets, recent efforts have focused on multi-omic profiling of patients. One such example comes from the Fudan University Shanghai Cancer Center (FUSCC), that profiled over 465 Chinese TNBC patients [109]. Initially, researchers identified numerous subgroups within the cohort and demonstrated that their transcriptomic subtyping best explained clinical features. Accordingly, the FUSCC-TNBC cohort was classified into four subtypes: Luminal Androgen Receptor (LAR), immunomodulatory (IM), basal-like immune-suppressed (BLIS), and mesenchymal-like (MES) [109]. This was subsequently followed up with the FUTURE trial that used the enrichment of certain transcriptomic signatures and genomic mutations within each subgroup to inform on which targeted therapies patients were most likely to respond to. The IM and MES subtypes showed satisfactory objective response rates (ORRs) to targeted therapies, the latter of which achieved over 50% ORR. However, the two other subgroups, LAR and BLIS, did not meet the expectations and showed ORRs under 20% [110].

Given that the initial analysis did not lead to good response rates for some TNBC subtypes, the authors expanded their study by integrating their transcriptomic subtyping with metabolomics data derived from LC-MS. In particular, they focused on finding metabolic vulnerabilities in the LAR and BLIS subtypes. To this end, 594 polar metabolites and 1944 lipids were profiled for 330 TNBC samples and 149 paired normal breast tissues, making this one of the biggest metabolic atlases created for TNBCs [111]. Using the similarity fusion network method, the authors divided samples into three clusters: C1, which possessed upregulations in sphingolipid and fatty acid metabolism and was predicted to have an increased dependence on fatty acids for energetic requirements; C2, which possessed an upregulation of glutamate pathways and, hence, was likely to rely more on glutamate metabolism; and C3, which possessed only small metabolomic differences compared to normal tissue.

Subsequently, the authors investigated the correlation between the metabolic subtypes and their previous transcriptomics classification. In particular, the C1 subtype showed a strong correlation with the transcriptomic LAR subtype. The LAR TNBC subtype showed increased levels of ceramides; this was validated using tracing experiments that highlighted the increased de novo synthesis and degradation of ceramides consistent with the C1 subtype’s increased reliance on fatty acid metabolism. To reveal potential metabolic vulnerabilities, the authors used inhibitors of every step in the ceramide biosynthesis pathway and demonstrated that SPHK1 inhibition was most effective in reducing the viability of LAR patient-derived organoids compared to non-LAR models. This approach highlights how metabolomics can complement other omics techniques to highlight specific vulnerabilities.

The association of the other TNBC transcriptomics subtypes with the metabolomics subtypes was less straightforward. However, using computational methods, the authors were able to establish that BLIS tumours that overlapped with the C2 metabolic subtype (BLIS-C2) showed a worse relapse-free survival compared to BLIS-C3 tumours. Using LASSO regression followed by a support vector machine classifier, the authors were able to distinguish BLIS-C2 from BLIS-C3 tumours based on the abundance of as few as six metabolites, highlighting a potential role for metabolomics in predicting patient outcomes. Using a similar approach as the one used for the LAR tumours, and focusing on BLIS-C2 with worse outcomes, the authors were able to identify NAAG as a metabolite of interest. RIMKLB, an enzyme that produces NAAG, was shown to be highly expressed in BLIS tumours and positively correlated with NAAG levels in the TNBC cohort. shRNA-mediated inhibition of RIMKLB led to a significant reduction in cancer cell proliferation, migration and invasion both in vitro, and in vivo that could be rescued by NAAG supplementation. RIMKLB, therefore, presents a novel target that can be used to treat the BLIS subtype, which is otherwise refractory to most therapies.

To further investigate metabolic vulnerabilities in TNBC subtypes, the authors integrated the multi-omics data of their cohort to develop a ferroptosis atlas. Ferroptosis is an iron-dependent form of cell death driven by an overload of lipid peroxides on cellular membranes and has attracted considerable attention as it may be used to treat tumours resistant to chemotherapy or other targeted therapies. A critical feature of ferroptosis is its execution via phospholipid peroxidation, which can be catalysed by lipoxygenases relying on iron, reactive oxygen species (ROS), and phospholipids containing primarily polyunsaturated fatty acid chains. On the other hand, cells possess three primary defence mechanisms that allow them to escape ferroptosis through the detoxification of lipid hydroperoxides via the action of the glutathione peroxidase 4 (Gpx4), lipophilic antioxidants like coenzyme Q10 (CoQ10), and thioredoxin reductase, which directly reduces lipid hydroperoxides by NADPH and selenocysteine. Targeting these escape mechanisms could, therefore, be used to treat tumours that have high rates of lipid peroxides.

Using an integrated transcriptomic and metabolomic approach, the authors were able to demonstrate the LAR subtype had multiple pathways shown to be upregulated that are related to ferroptosis, including fatty acid synthesis, ROS accumulation, and glutathione (GSH) metabolism. LAR tumours had higher rates of lipid peroxidation and were shown to upregulate the SLC7A11/GSH/GPX4 axis to escape ferroptosis. Using various pharmacological inhibitors for ferroptosis suppressors, the authors were able to show that targeting GPX4, that catalyses the oxidation of GSH to GSSG, through the inhibitor RSL3 led to much greater cell death in LAR tumours compared to other TNBC subtypes. Interestingly, the authors showed that GPX4’s expression was dependent on androgen receptor (AR) signalling, but treating the tumours with AR inhibitors did not prime LAR tumours for ferroptosis. This is likely due to the plural role of AR in ferroptosis, with high AR expression leading to 2,4-Dienoyl-CoA Reductase 1 (DECR1) downregulation, whose knockdown selectively inhibits β-oxidation of PUFAs that serve as substrates for lipid peroxidation. As a result, for GPX4, but not AR, inhibition led to ferroptosis in LAR tumours, highlighting the importance of this specific metabolic target. The authors further showed that GPX4 inhibition in vivo led to the reprogramming of the tumour microenvironment and increased the infiltration of CD8+ T cells and M1 macrophages that exert anti-tumour effects. The authors thus suggested that a novel combination therapy of ferroptosis inducers and immune checkpoint inhibitors (ICIs) could show synergy to treat LAR tumours and significantly improve patient outcomes.

Overall, these studies underscore the value of performing metabolomics alongside other omics profiling technologies. While transcriptomics has led to better stratification of TNBCs, there is still an unmet clinical need to predict therapies that these tumours would respond to. Using metabolomics, the authors were able to uncover new metabolic vulnerabilities that showed promising pre-clinical applications and demonstrated that metabolic therapies can also be used to potentiate other forms of treatment, such as ICIs. This case study demonstrates the value of a multimodal pipeline for the treatment of refractory TNBCs.

### 4.2. Glioblastomas

Glioblastoma multiforme (GBM) is one of the most lethal and most common subtypes of central nervous system tumours and is often associated with very poor survival rates [112]. Much work has gone into studying GBM’s genomes and transcriptomes and yielded a better understanding of molecular alterations that drive GBMs, including alterations in the core RTK/RAS/PI3K, p53 and RB signalling pathways [113]. Previous profiling has led to GBM’s stratification into four transcriptomic subtypes, namely classical, mesenchymal, neural, and proneural [114]. GBMs are also classified based on their IDH1/2 mutation status [115]. While studies have led to better knowledge of GBM drivers, GBMs display extensive genetic and transcriptomic heterogeneity that limit the efficacy of specific targeted therapies [116]. This has led to the majority of glioblastomas being incurable with current treatment options, highlighting an urgent clinical need to identify more effective therapeutic strategies for these tumours [117].

Similarly to the work conducted for TNBC, multi-omics cohorts for GBMs have leveraged metabolomics to better understand GBM progression and discover metabolic vulnerabilities that can be used for treatment. One such study comes from Minami et al. that integrated genomics, transcriptomics, and lipidomics from a diverse GBM cohort consisting of patient samples, orthotopic xenografts (PDXs), and gliomaspheres (GS) cell cultures [118]. Using shotgun lipidomics, the authors were able to identify 1020 different lipid species from 15 lipid subgroups, making this one of the largest lipidomics studies performed for GBMs. They were able to separate the metabolic species into four lipid groups that contained species representing specific subclasses and FA saturation: L1, that mostly consisted of triacylglycerols (TAGs) with monounsaturated fatty acids (MUFAs), L2, which consisted of TAGs with polyunsaturated fatty acids (PUFAs), L3, which contained various phospholipid species, including phosphatidylcholines (PCs), phosphatidylethanolamine (PEs), free fatty acids (FFAs) and diacylglycerols (DAGs), and L4, which primarily consisted of ether lipids and ceramides. These lipid clusters had a strong correspondence with gene-related metabolic signatures based on gene ontology enrichment, showing a strong transcriptomic–metabolic relationship.

The authors were then able to leverage this relationship to identify cell-intrinsic regulators of GBM metabolism. They identified that CDKN2A, which was deleted in up to 60% of GBMs, had an important role in tail length, and the saturation state of a significant subset of lipids in all the aforementioned lipid subgroups. More specifically, the authors showed that CDKN2A governed the partitioning of oxidizable PUFAs into TAGs where they were protected from lipid peroxidation. As CDKN2A null tumours lacked this ability, they had highly desaturated TAGs with shorter fatty acid tails. This led to an increase in the proportion of oxidizable PUFAs in phospholipids in the cellular membrane, and as a result, higher rates of lipid peroxidation. This primed CDKN2A null GBMs for ferroptosis and inhibition of GPX4 through RSL3 led to significant cell death in CDKN2A null but not CDKN2A WT tumours. This vulnerability was recapitulated in vivo through mouse models that showed that GPX4 inhibition led to prolonged survival in CDKN2A null, but not CDKN2A WT GBM tumours. Importantly, the authors were able to show that this trend was captured in primary tumours, but not cell lines, providing further evidence for the importance of conducting metabolomics in patient cohorts.

Overall, these studies highlight the importance of performing metabolomics studies in large patient cohorts. In the case of GBMs, metabolomics allowed for a better understanding of tumour progression and helped discover previously unknown roles of key cancer drivers, such as CDKN2A’s role in PUFA partitioning, while in TNBCs, it was shown that it can be used to better predict survival in patients, such as the BLIS transcriptomic subtype. Importantly, it also helped to unveil novel metabolic vulnerabilities that can be used to treat tumours refractory to therapies, as is the case for GPX4 inhibition in tumours with high rates of lipid peroxidation. These case studies thus highlight the multi-faceted role of metabolomics in patient cohorts (Table 2).

## 5. Future Directions

Metabolomics has shown extensive applications for diagnosing and treating tumours but it is most often used in isolation and is not always integrated with other profiling modalities, such as spatial omics or proteomics. Further advances in metabolomics and computational techniques will allow for a more holistic understanding of tumour biology. This section briefly looks at some analytic techniques that show promise in their application to cancer precision medicine.

### 5.1. Using Multi-Omics Tools and Machine Learning to Examine Tumour Metabolism and Predict Therapy Response

Multi-omics methods have been applied pre-clinically through cell lines and mouse models to derive many insights about tumour metabolism and identify novel metabolic vulnerabilities. Pan-cancer analyses in cell lines have linked tumour metabolic changes to specific genetic alterations, epigenetic features, or transcriptional dependencies. This study unveiled genomic factors driving tumour metabolic reprogramming, including the amplification of the malic enzyme 2 and the proposed targeting of asparaginase metabolism in gastric cancer. Another study integrated metabolomics in cell lines with proteomics and transcriptomics to uncover transcriptional regulators of metabolism and crosstalk between the metabolic and transcriptional levels. In particular, they uncovered a relationship between glucose and one-carbon metabolism, suggesting that tumours that have lower glucose uptake rates could be sensitive to anti-folate drugs. This computational framework can exploit tumour metabolic profiles to find appropriate transcriptional regulators that underly targetable metabolic dysregulations. While these studies have shed light on novel metabolic targets, as evidenced in the glioblastoma study in Section 4, there is still discord between the applicability of tumour metabolic targets pre-clinically and in patients. Accordingly, multi-omics studies, such as those outlined in Section 4, must be performed to gain more meaningful and physiologically relevant insights.

In cases where metabolomics data are available for patients, machine learning (ML) can aid in using the data to classify patients based on response to therapies or specific phenotypes of interest. LASSO regression has been used extensively to reduce dimensional space and reduce the number of metabolites associated with predictions of phenotypes, such as therapy response or cancer staging. This allows for more targeted metabolic panels to be applied to ML algorithms, such as support vector machines, random forest, or deep learning classifiers. ML and multi-omics can further converge through building boosted classifiers. Boosted classifiers are composed of two or more sub-classifiers that make predictions based on their respective information modalities. Predictions from each classifier can then be integrated to make better predictions. An example of this was applied recently to predict radiation responses in tumours that used a boosted classifier. One classifier was based on a targeted metabolite panel developed earlier, mostly comprising redox metabolites, and another was based on a transcriptomics-based classifier. Integrating both classifiers significantly improved the performance of the classifier, which was able to achieve a sensitivity of over 90%, underscoring the power ML and multi-omics possess when integrated.

### 5.2. Analytic Techniques Can Aid in Discovering Metabolic Biomarkers or Vulnerabilities

It may not always be possible to perform untargeted metabolomics due to cost or the inability to profile desirable subsets of the metabolome. In this case, analytic techniques, such as FBA, can be used to study metabolic reprogramming to hone in on specific pathways or metabolic targets. FBA’s major advantage is its ability to generate metabolic fluxomic data from just transcriptomic data. With the advent of a more complete reference genome-scale metabolic models, such as Human1, FBA’s accuracy in predicting human metabolic reprogramming has greatly improved over the past few years. A recent example of the application of FBA to study tumour metabolic reprogramming was to study the metabolism of TNBC metastases. In particular, breast cancer metastases upregulated both oxidative phosphorylation and glycolysis compared to the matched primary tumours to increase metabolic flexibility in harsher nutrient conditions. FBA also revealed an increase in transport reaction flux, indicating metastases take up more nutrients from the microenvironment rather than synthesise de novo, owing to the scarcity of some nutrients in the microenvironment. Further applications of FBA can be used to bridge the gap between metabolomics and other profiling technologies.

### 5.3. Single-Cell and Spatial Metabolomics Technologies

Single-cell and spatial technologies have enabled unprecedented studies into understanding tumour heterogeneity and the role of the tumour microenvironment. However, in the past few years, single-cell omics has primarily focused on genomics, transcriptomics and proteomics, while metabolomics has lagged. However, recent technological developments hold promise in enabling the performance of single-cell metabolomics. One such framework, ScSpaMet, has recently been developed to allow highly multiplex untargeted metabolomic imaging on tumour tissue. In this framework, cells are labelled with metal-isotope conjugated antibodies and metabolic profiling is performed by 3D-SMF [119]. Combining this with spatial proteomics approaches, the authors characterised the tumour metabolic environment of lung tumour microarrays. Accordingly, they were able to identify differences in metabolism between tumour and stromal-associated regions, including differences in glycolysis and cholesterol metabolism. While this approach is limited to profiling a few metabolic fragments, further advances in metabolite annotation in mass spectra will allow for more detailed profiling of tumour metabolic heterogeneity to complement current MSI approaches.

## 6. Conclusions

Metabolic reprogramming in cancer has only recently been acknowledged as a significant hallmark, and it is often overlooked in favour of other hallmarks, such as altered signalling pathways or the importance of escape from the immune system. This is in large part due to the lack of a singular technology to profile whole-cell metabolomics, unlike for other omics technologies such as genomics and transcriptomics. However, by combining different technologies, it is possible to examine a large subset of the tumour metabolome and focus on relevant pathways. When metabolomic techniques are applied to study tumour biology, they can unveil biomarkers and targeted therapies not achievable by other profiling technologies. Metabolic imaging can be used to sensitively detect and monitor response to therapy non-invasively, while plasma and serum metabolomics allow detailed diagnostic and prognostic information to be gathered easily from patients. Metabolic profiling of tumour samples allows a detailed understanding of tumour heterogeneity and can reveal new clinical targets that can better treat subtypes of tumours that are often intractable to therapy, such as glioblastomas or TNBCs. Successful integration of metabolomics with other omics technologies will allow clinicians to further advance personalised metabolic biomarkers and identify effective therapies for patients with cancer.

## Figures and Tables

**Table 1:** Overview of the advantages and disadvantages of different metabolomics techniques.

Technique	Advantage	Disadvantage
GC-MS	Quantitative (with appropriate standards and calibration). Good sensitivity in metabolite detection. Good reproducibility in separation of compounds. Detects most organic and some inorganic molecules. Extensive support and use mean many programmes and libraries assist in metabolite identification.	Requires sample derivatization. Metabolites in their solid form cannot be used. Relatively slow (20–40 min per sample). Novel compound identification is difficult.
LC-MS	Quantitative (with appropriate standards and calibration). Excellent sensitivity for metabolite detection. Can detect a large proportion of the metabolome. Detects most organic and some inorganic molecules. Extensive support and use mean many programmes and libraries assist in metabolite identification. Potential to pair with other technologies to obtain more modes of information.	Instruments and running them are costly. Relatively slow (20–40 min per sample). Not compatible with gases. Relatively low reproducibility between different instruments and analysis prone to batch effects. Poor separation resolution compared to GC-MS. Novel compound identification is difficult.
Metabolic imaging—PET	Non-invasive detection of tumours. High sensitivity for detection of radiotracers and changes in levels. Whole-body imaging allows cancer staging and metastases detection. Can be used to monitor tumour response to therapy. Allows early detection of tumours before structural abnormalities appear.	Low spatial resolution for detection of location of tumour. Limited species can be used for PET scans. False positives generated based on non-specific tracer uptake. Tracers can be costly.
Metabolic Imaging—MRS	Preserves spatial information. DNP means that metabolites can be detected at low concentrations. Non-invasive form of imaging. Can develop tracers specific to specific cancer’s metabolism, e.g., lipid tracers for prostate cancer.	Low sensitivity and limited spatial resolution. Long acquisition times. Spectral overlap from different metabolites makes quantification difficult.
Mass Spectrometry Imaging	Multiplexed analysis allows detection of many metabolites. Provides spatial metabolomics information. Label free imaging. Provides semi-quantitative levels of metabolites.	Requires biopsy from patient and complex slide preparation. Limited metabolome coverage. Ion suppression effects from matrix can make relative quantification inaccurate.
Extracellular Flux Analysis	Simultaneous measure of glycolysis and oxidative phosphorylation rates.	Indirect measurement of oxidative phosphorylation and glycolytic rates.

**Table 2:** Summary of metabolomics applications used in two case studies.

Category	Fudan Cohort	Glioblastomas
Type of cancer	Triple-negative breast cancer (TNBC)	Glioblastomas
Metabolomics performed	594 polar metabolites 1944 lipids	1020 lipid species from 15 distinct lipid groups
Metabolomic subtypings	C1: Upregulations in sphingolipid and fatty acids; increased dependency on fatty acids C2: Upregulation and increased dependency on glutamate metabolism C3: Comparatively reduced metabolic reprogramming and like normal tissue	L1: upregulations of triacylglycerols (TAGs) with monounsaturated fatty acids (MUFAs) L2: upregulation of TAGs with polyunsaturated fatty acids (PUFAs) L3: upregulation in phospholipid species including phosphatidylcholines (PCs), phosphatidylethanolamine (PEs), free fatty acids (FFAs) and diacylglycerols (DAGs) L4: upregulation in ether lipids and ceramides
Metabolite-transcriptomic associations	Transcriptomic LAR subtype associated with C1 BLIS subtype associated with C2 and C3 differentiated by LASSO regression LAR tumours upregulated the SLC7A11/GSH/GPX4 axis	Strong association of metabolic subtypes with gene ontology classes CDK2NA controlled partitioning of oxidizable PUFAs into TAGs L1 subtype had higher proportion of CDKN2A null tumours and L2 had lower proportion
Therapeutic insights gained	SPHK1 inhibition effective in LAR subtype as targets ceramide biosynthesis RIMKLB is novel target in BLIS subtype LAR subtype sensitive to ferroptosis inhibitor RSL3 Treatment of LAR tumours by RSL3 led to increased immune infiltration; suggests synergism between RSL3 and ICI	CDK2NA null tumours sensitive to ferroptosis inhibition by RSL3 Showed differences in sensitivity between tumour samples and cell lines

## Data Availability

No new data were created.
